# Preventable Diabetic Complications After a Cancer Diagnosis in Patients With Diabetes: A Population-Based Cohort Study

**DOI:** 10.1093/jncics/pky008

**Published:** 2018-05-11

**Authors:** Erin Worndl, Kinwah Fung, Hadas D Fischer, Peter C Austin, Monika K Krzyzanowska, Lorraine L Lipscombe

**Affiliations:** 1 Department of Medicine, University of Toronto, Toronto, ON, Canada; 2 Institute for Clinical Evaluative Sciences, University of Toronto, Toronto, ON, Canada; 3 Institute of Health Policy, Management and Evaluation, University of Toronto, Toronto, ON, Canada; 4 Department of Medical Oncology, Princess Margaret Hospital, Toronto, ON, Canada; 5 Department of Medicine, Women’s College Research Institute, Women’s College Hospital, Toronto, ON, Canada

## Abstract

**Background:**

A cancer diagnosis may disrupt diabetes management, increasing the risk of preventable complications. The objective was to determine whether a cancer diagnosis in patients with diabetes is associated with an increased risk of diabetic complications.

**Methods:**

This retrospective cohort study using health care data from Ontario, Canada, included persons age 50 years or older diagnosed with diabetes from 2007 to 2011 and followed until 2014. We examined the effects of cancer as a time-varying covariate: breast cancer (in women), prostate cancer (in men), colorectal cancer, and other cancers (in men and women). Each cancer exposure was categorized as stage I–III, IV, or unknown, and by time since cancer diagnosis (0–1 year, >1–3 years, and >3 years). The primary outcome was hospital visits for diabetic emergencies. Secondary outcomes were hospital visits for skin and soft tissue infections and cardiovascular events.

**Results:**

Of 817 060 patients with diabetes (mean age = 64.9 +/- 10.7 years), there were 9759 (1.2%) colorectal and 45 705 (5.6%) other cancers, 6714 (1.7%) breast cancers among 384 257 women and 10 331 (2.4%) prostate cancers among 432 803 men. For all cancers except stage I–III prostate cancer, rates of diabetic complications were significantly higher zero years to one year after diagnosis compared with no cancer (adjusted relative rates ranging from 1.26, 95% confidence interval [CI] = 1.08 to 1.49, to 4.07, 95% CI = 3.80 to 4.36); these differences were attenuated in the subsequent periods after cancer diagnosis.

**Conclusions:**

Patients with diabetes are at increased risk for preventable complications after a cancer diagnosis. Better diabetes care is needed during this vulnerable period.

Diabetes affects up to one-third of patients with cancer (1) and is associated with an increase in cancer incidence and a 40% higher mortality rate following cancer diagnosis (2,3). Diabetes has effects on both cancer-specific and noncancer mortality (3,4). For example, the metabolic effects of excess glucose and insulin have been shown to promote tumor growth and aggressiveness (5,6). Additionally, patients with diabetes have lower rates of cancer screening (7,8) and more advanced-stage cancers at diagnosis (9). Patients with comorbidities such as diabetes may also be treated less aggressively for cancer due to higher risks of toxicity (10,11).

Diabetes-related complications may also contribute to higher noncancer mortality in diabetes patients with cancer. As cancer survival continues to improve, comorbid conditions such as diabetes can have a greater impact on life expectancy and quality of life than the initial cancer. While patients with diabetes are at increased risk for metabolic and vascular complications, it is uncertain whether having cancer further exacerbates that risk. Some studies have examined how diabetes management is affected by a cancer diagnosis, postulating that cancer-related services may compete with delivery of primary care for diabetes. There has been conflicting evidence, whereby some studies found that frequency of glycated hemoglobin (HbA1c) measurements decreased (12,13) while others found no change or improvement in monitoring and control (14,15) after a cancer diagnosis. Studies have also shown that adherence to diabetes medications, statins, and antihypertensives decreases after a cancer diagnosis in diabetes patients (16–18).

A recent study from the United Kingdom evaluated long-term microvascular and macrovascular outcomes among patients with preexisting diabetes after a cancer diagnosis, and risks were similar to patients without cancer (19). However, no study to our knowledge has evaluated the impact of cancer on preventable short-term diabetic complications. The period after a cancer diagnosis can be associated with enhanced stress and demanding treatment regimens, which may disrupt management of comorbid conditions as cancer treatment is prioritized. For patients with diabetes, the lack of timely concurrent diabetes support may place patients at higher risk for destabilized metabolic control and potentially avoidable acute complications. The Centers for Disease Control and Prevention considers diabetes to be an ambulatory care sensitive condition, whereby serious complications and hospitalizations are potentially preventable with effective outpatient management (20). Such consequences not only affect diabetes outcomes but may also limit cancer treatment protocols and worsen cancer survival. In that context, the objective of our study was to examine whether a new cancer diagnosis in patients with diabetes is associated with an increased risk of preventable diabetic complications, and how this risk changes with time after cancer.

## Methods

### Study Design and Setting

We conducted a population-based, retrospective cohort study between January 1, 2007, and March 31, 2014, in Ontario, Canada, to compare the rate of acute diabetic complications after a cancer diagnosis with the time period without cancer in persons with diabetes.

### Data Sources

We used health administrative databases, which include records for virtually all Ontario residents through coverage under the universal Ontario Health Insurance Plan (OHIP). Outcomes were identified using the Hospital Discharge Abstract Database from the Canadian Institute for Health Information (CIHI), which contains data on up to 25 International Classification of Disease (ICD)–10 diagnosis codes, and the National Ambulatory Care Reporting System to identify primary diagnoses from emergency department (ED) visits. Diabetic complications were captured based on the diagnosis most responsible for the hospital stay (type 1), which accurately identifies approximately 99% of cases based on quality audits at our institution. Demographic and comorbidity data were obtained from those databases, the Registered Persons Database, and the OHIP Physicians Claim Database. Patients with diabetes were identified from the validated Ontario Diabetes Database, based on two physician visits or one hospitalization for diabetes within two years (21). We used the Ontario Cancer Registry (OCR) to detect cancer cases recorded since 1964, which contains data for more than 90% of cancers diagnosed in Ontario, based on records from hospitals, regional cancer centers, pathology reports, and death certificates (22). These data sets were linked using unique encoded identifiers and analyzed at the Institute for Clinical Evaluative Sciences (ICES).

### Study Population and Cohort Definition

The study population included persons age 50 to 105 years with prevalent diabetes (at least two years) between January 1, 2007, and March 31, 2011. We excluded patients younger than age 50 years, who are more likely to have genetically related cancers, and those without at least two years of health insurance, those with a previous diagnosis of cancer, or those living in a long-term care facility. We derived three separate cohorts based on sex to account for cancers that are sex-specific: women only, men only, and men and women (combined). Cohort entry was defined as the date when both the age and diabetes criteria were met.

### Cancer Exposure

We used the OCR to identify the first new cancer diagnosis within each cohort from cohort entry until March 31, 2011, treating cancer as a time-varying covariate. We identified four cancer exposure categories: breast (in the women only cohort), prostate (in the men only cohort), colorectal (in the combined men and women cohort), and other cancers (men and women). We isolated breast, prostate, and colorectal cancers as they are common, include more complete cancer stage data, have highly variable cancer treatment courses, and have relatively good prognoses where ongoing management of comorbidities would be important. We categorized each cancer exposure as stage I–III, IV, or unknown (missing or uncertain) to account for potential differences in cancer prognosis and diabetes care needs, and further subdivided by time since cancer diagnosis: zero to one year, more than one to three years, and more than three years postcancer. We compared each cancer category to a common noncancer reference category (patients without cancer and the time before diagnosis in those with cancer). Subjects were followed from date of cohort entry. For example, prior to the date of colorectal cancer diagnosis, subjects were classified as unexposed. From the date of colorectal cancer diagnosis onwards, exposure was categorized by stage and further subdivided into periods after diagnosis in a time-dependent fashion. The effect of each exposure category (eg, colorectal cancer stage I–III, 0–1 year postcancer) was compared with the noncancer time period within the same cohort. We used similar methods to categorize other cancers within the combined cohort and to categorize breast cancers in women and prostate cancers in men.

### Study Outcomes and Follow-up

Our primary outcome was time to hospital visits (emergency department visits or hospital admissions) for diabetic emergencies, where the main or most responsible diagnosis (type 1) was diabetes, hypoglycemia, hyperglycemia, diabetic ketoacidosis, or hyperosmolar state. Secondary outcomes included hospital visits for skin and soft tissue infections (diabetic foot infections, cellulitis, skin abscess, and gangrene) and cardiovascular events (a main diagnosis of acute myocardial infarction, congestive heart failure, or stroke). We also examined process measures of diabetes management: frequency of primary care diabetes visits and dilated eye examinations (14). Patients were followed for outcomes from cohort entry until the first of the following: death, emigration from Ontario, development of a second cancer, the end of the study period, or March 31, 2014.

### Baseline Covariates

Demographic covariates included age, sex, region of residence, and neighborhood income quintile based on postal codes determined at cohort entry. We also assessed comorbidity burden using the John’s Hopkins Aggregated Diagnosis Group (ADG) comorbidity score (23). We measured health service use based on hospitalizations for cardiovascular disease, hypoglycemia, hyperglycemia, and skin infections in the previous five years; at least one dilated retinal exam in the previous two years, and at least one primary care diabetes visit in the previous year.

### Statistical Analysis

For each of the outcomes listed above, we conducted time-to-event analyses using extended Cox regression models for recurrent outcomes to estimate associations between cancer exposures and outcomes, which allowed us to capture all of the events contributed by each patient within each time period (24). We conducted separate analyses for each of the four cancer cohorts and for each outcome of interest. We expressed the frequency of events within a time period as a crude number of events and a rate per 100 person-years (PY). We compared rates of events in the noncancer period to each cancer exposure within their relevant cohorts and expressed these as relative rates, unadjusted and adjusted for age, sex (for the combined cohort), diabetes duration, weighted ADG score, neighborhood income quintile, and rural residence.

## Ethics

This study was approved by the Research Ethics Board of Sunnybrook Hospital.

## Results

### Baseline Characteristics

**Figure 1.**
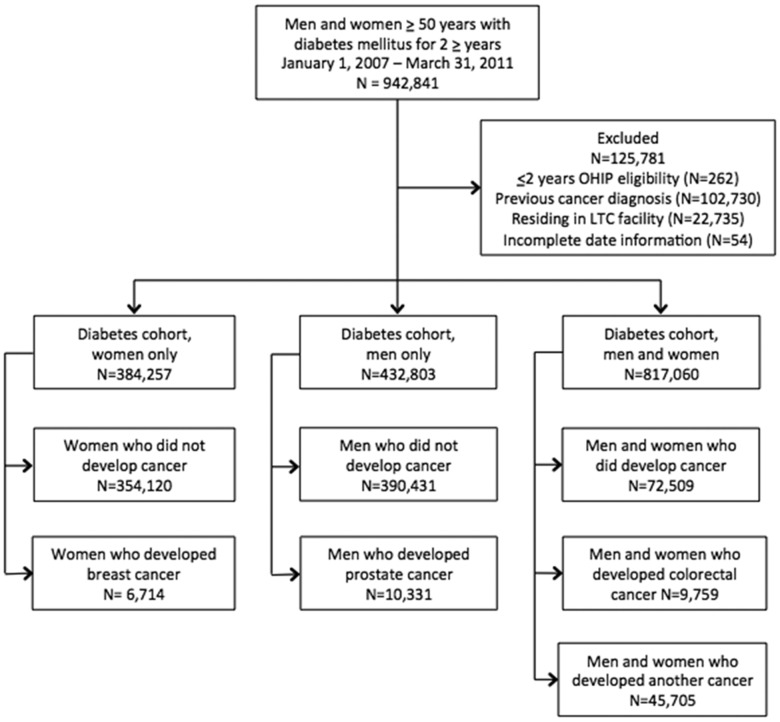
Description of the cohort selection of persons with diabetes, with and without a cancer diagnosis. LTC = Long-term care facility; OHIP = Ontario Health Insurance Plan.

Within the 817 060 men and women with diabetes who met the inclusion criteria, there were 9759 (1.2%) colorectal and 45 705 (5.6%) other cancers during a mean 5.9 years of follow-up, and there were 6714 (1.7%) breast cancers among 384 257 women and 10 331 (2.4%) prostate cancers among 432 803 men (Figure 1). The “other cancers” category was comprised of lung (22.4%), urinary (12.1%), female genital (8.5%), lymphoma (7.1%), digestive (6.1%), pancreas (5.0%), leukemia (4.8%), melanoma (4.2%), gastric (3.8%), liver (3.4%), thyroid (3.1%), oropharyngeal (3.1%), and other (16.4%) cancers. In the combined cohort, the mean age was 64.9 +/- 10.7 years, and diabetes duration was 6.8 +/- 4.9 years at cohort entry; women had similar diabetes duration but were a mean two years older than men (Table 1).

**Table 1.** Characteristics of persons with diabetes in Ontario, Canada, between age 50 and 105 years

Characteristic	Female patients (n = 384 257)	Male patients (n = 432 803)	All patients (n = 817 060)
Age, mean (SD), y	66.0 (11.2)	64.0 (10.2)	64.9 (10.7)
Males, No. (%)	0 (0.0)	432 803 (100.0)	432 803 (53.0)
Rural residence, No. (%)	4455 (1.2)	5907 (1.4)	10 362 (1.3)
SES*, No. (%)			
1	91 920 (23.9)	89 266 (20.6)	181 186 (22.2)
2	85 057 (22.1)	91 001 (21.0)	176 058 (21.5)
3	74 357 (19.4)	85 568 (19.8)	159 925 (19.6)
4	68 310 (17.8)	83 421 (19.3)	151 731 (18.6)
5	58 600 (15.3)	75 983 (17.6)	134 583 (16.5)
Diabetes duration, mean (SD), y	6.8 (4.9)	6.7 (4.8)	6.8 (4.9)
Weighted ADG score† (SD)	12.0 (11.5)	12.0 (11.8)	12.0 (11.6)
Past medical history, No. (%)			
Cardiovascular disease‡	46 247 (12.0)	68 354 (15.8)	114 601 (14.0)
Hypoglycemia§	485 (0.1)	500 (0.1)	985 (0.1)
Hyperglycemia‖	1260 (0.3)	1424 (0.3)	2684 (0.3)
Hospitalization for diabetes	6812 (1.8)	9266 (2.1)	16 078 (2.0)
Skin infections	2124 (0.6)	3382 (0.8)	5506 (0.7)
Eye exam	203 601 (53.0)	210 812 (48.7)	414 413 (50.7)
Physician visit for diabetes management	108 497 (28.2)	127 334 (29.4)	235 831 (28.9)
First cancer diagnosis in study period, No. (%)			
Colorectal	3947 (1.0)	5812 (1.3)	9759 (1.2)
Breast	6714 (1.7)	0 (0.0)	6714 (0.8)
Prostate	0 (0.0)	10 331 (2.4)	10 331 (1.3)
Other	19 476 (5.1)	26 229 (6.1)	45 705 (5.6)
None	354 120 (92.2)	390 431 (90.2)	744 551 (91.1)
Cancer stage, No. (%)			
1	6686 (1.7)	5 021 (1.2)	11 707 (1.4)
2	4724 (1.2)	9282 (2.1)	14 006 (1.7)
3	3966 (1.0)	5012 (1.2)	8978 (1.1)
4	4081 (1.1)	6561 (1.5)	10 642 (1.3)
Unknown¶	10 680 (2.8)	16 495 (3.8)	27 175 (3.3%)

* Socioeconomic status, as defined by the neighborhood income quintile. SES = socioeconomic status.

† John’s Hopkins comorbidity score, two-year look-back window.

‡ Cardiovascular disease defined as composite of hospitalization for acute myocardial infarction, congestive heart failure, and stroke, five-year look back.

§ Previous hospitalization for hypoglycemia, five-year look back.

‖ Previous hospitalization for hyperglycemia, five-year look back.

¶ Stage data unavailable in the Ontario Cancer Registry (not recorded in the database).

### Rates of Diabetic Emergencies

**Figure 2.**
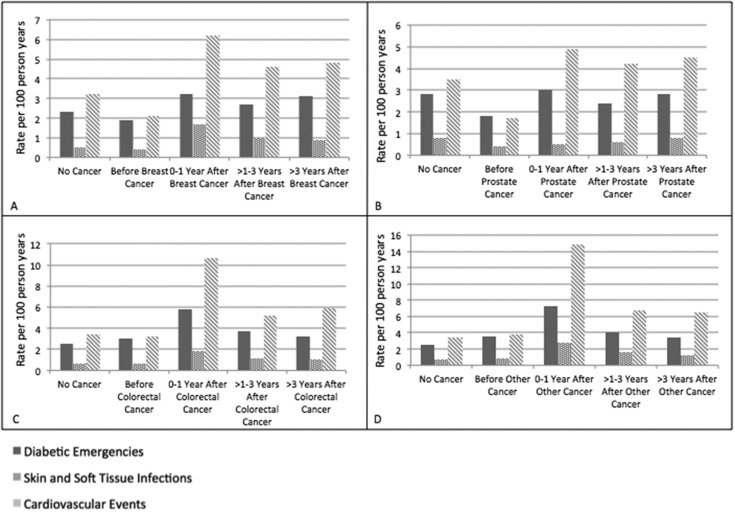
Rates of the outcomes of interest (diabetic emergencies, skin and soft tissue infections, and cardiovascular events) among patients without cancer and among patients who developed **(A)** breast cancer; **(B)** prostate cancer; **(C)** colorectal cancer; and **(D)** other cancer, expressed in rate per 100 person-years. For the graphs showing the rates of the outcomes of interest among patients with colorectal cancer **(C)** and other cancer **(D)**, the cohort of patients included all men and women, whereas for breast cancer **(A)**, the cohort of patients included only women, and for prostate cancer **(B)**, the cohort of patients included only men.

The cumulative rate of diabetic emergencies was 2.5 per 100 PY among patients who never developed cancer during the entire study period. Among those who developed cancer, cumulative rates of diabetic emergencies before the development of cancer were 1.8 per 100 PY in men who developed prostate cancer, 1.9 per 100 PY in women who developed breast cancer, and 3.0 and 3.5 per 100 PY in men and women who developed colorectal and other cancers, respectively. In all four cohorts, cumulative rates of diabetic emergencies increased 1.5- to twofold within the first year after cancer diagnosis (Figure 2). Rates declined during one to three years and more than three years after diagnosis, though they remained higher than the precancer period. Trends in event rates for individual cancer sites within the “other cancer” category were generally similar to those reported for the entire category (data not shown).

**Figure 3.**
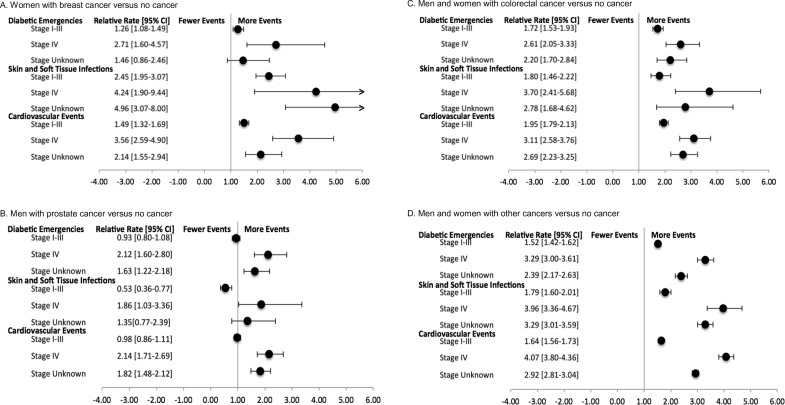
Adjusted relative rates of diabetic emergencies, skin and soft tissue infections, and cardiovascular events in patients diagnosed within the first year after cancer diagnosis compared with patients without cancer: **(A)** breast cancer (women); **(B)** prostate cancer (men); **(C)** colorectal cancer (men and women); and **(D)** other cancers (men and women). **Data points** represent relative rates calculated for each cancer stage category, with **bars** representing 95% confidence intervals. CI = confidence interval.

**Figure 4.**
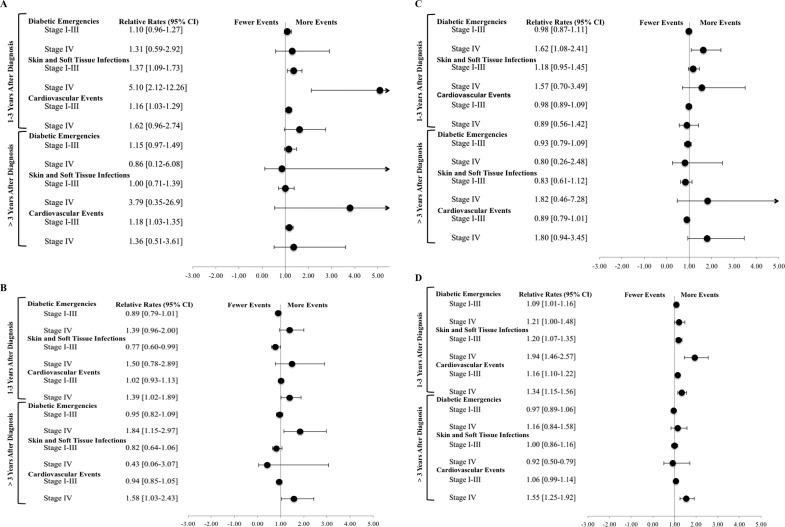
Adjusted relative rates of diabetic emergencies, skin and soft tissue infections, and cardiovascular events in patients diagnosed within the second and third years after cancer diagnosis compared with patients without cancer: **(A)** breast cancer (women); **(B)** prostate cancer (men); **(C)** colorectal cancer (men and women); and **(D)** other cancers (men and women). **Data points** represent relative rates calculated for each cancer stage category, with **bars** representing 95% confidence intervals. CI = confidence interval.

Figure 3 shows the adjusted relative rates of acute complications in the first year after cancer diagnosis for each cancer subtype compared with the noncancer period. After adjusting for covariates, the relative rates of diabetic emergencies during the first year after cancer diagnosis were statistically significantly higher than the noncancer period for all cancers (adjusted relative rates [aRRs] range from 1.26, 95% confidence interval [CI] = 1.08 to 1.49, for stage I–III breast, to 3.29, 95% CI = 3.00 to 3.61, for other stage IV cancers), except stage I–III prostate cancer. Relative rates were higher for stage IV than stage I–III cancers in all cohorts. Relative rates of diabetic emergencies remained statistically significantly elevated in the one to three years after cancer compared with the noncancer period for patients with stage IV colorectal and all stages of other cancers (Figure 4). No differences were found between the more-than-three-years postcancer and noncancer periods, except for in men with stage IV prostate cancer.

### Skin and Soft Tissue Infections and Cardiovascular Events

We found a statistically significant increase in the rate of skin and soft tissue infections and cardiovascular events in the first year after all cancer diagnoses except stage I–III prostate cancer (Figures 2 and 3). Compared with the noncancer period, women with breast cancer had a statistically significant 2.5- (stage I–III) to 4.2-fold (stage IV) higher rate of skin and soft tissue infections and 1.5- to 3.6-fold higher rate of cardiovascular events zero years to one year after diagnosis (Figure 3). We found similar trends for patients who developed colorectal and other cancers. As for diabetic emergencies, relative rates of other complications declined in the latter postcancer periods in all cohorts (Figure 4).

### Process Indicators of Diabetes Care

Table 2 summarizes the results for physician visits for diabetes management and eye examinations in the postcancer period. We found that for the majority of cancer patients, the rate of physician visits for diabetes management was lower compared with the noncancer period. There were no differences in eye examinations for stage I–III cancer patients, but rates were significantly lower in all stage IV cancer patients.

**Table 2.** Unadjusted and adjusted relative rates of visits for diabetes management and dilated eye examinations in patients at 0–1 years, >1–3 years, and >3 years after a cancer diagnosis relative to diabetes patients without cancer*

Type of visit	Cancer-free period	Time after cancer diagnosis, y					
		0–1		>1–3		>3	
		URR (95% CI)	ARR (95% CI)	URR (95% CI)	ARR (95% CI)	URR (95% CI)	ARR (95% CI)
Diabetes management							
Breast cancer							
Stage I–III	1.0	0.86 (0.82 to 0.90)	0.87 (0.83 to 0.91)	0.95 (0.91 to 0.99)	0.97 (0.93 to 1.01)	0.90 (0.85 to 0.96)	0.93 (0.88 to 0.99)
Stage IV	1.0	0.59 (0.45 to 0.77)	0.61 (0.46 to 0.79)	0.57 (0.42 to 0.79)	0.60 (0.44 to 0.82)	0.17 (0.06 to 0.52)	0.19 (0.06 to 0.57)
Stage unknown	1.0	0.82 (0.68 to 0.99)	0.83 (0.69 to 1.01)	0.83 (0.69 to 0.99)	0.85 (0.72 to 1.02)	0.74 (0.58 to 0.94)	0.78 (0.61 to 0.99)
Prostate cancer							
Stage I–III	1.0	0.95 (0.92 to 0.98)	0.96 (0.93 to 1.00)	0.94 (0.91 to 0.97)	0.97 (0.94 to 1.00)	0.92 (0.88 to 0.96)	0.96 (0.92 to 1.01)
Stage IV	1.0	0.84 (0.73 to 0.97)	0.87 (0.76 to 1.00)	0.91 (0.80 to 1.05)	0.95 (0.83 to 1.09)	0.80 (0.63 to 1.03)	0.84 (0.66 to 1.09)
Stage unknown	1.0	0.87 (0.79 to 0.97)	0.90 (0.81 to 0.99)	0.81 (0.74 to 0.88)	0.84 (0.77 to 0.92)	0.82 (0.74 to 0.92)	0.87 (0.78 to 0.97)
Colorectal cancer							
Stage I–III	1.0	0.85 (0.81 to 0.89)	0.88 (0.84 to 0.92)	0.94 (0.90 to 0.97)	0.97 (0.93 to 1.01)	0.87 (0.82 to 0.92)	0.92 (0.86 to 0.97)
Stage IV	1.0	0.51 (0.44 to 0.60)	0.51 (0.43 to 0.59)	0.71 (0.60 to 0.84)	0.72 (0.61 to 0.86)	0.76 (0.55 to 1.03)	0.80 (0.59 to 1.10)
Stage unknown	1.0	0.76 (0.66 to 0.87)	0.78 (0.68 to 0.89)	0.89 (0.79 to 1.01)	0.92 (0.81 to 1.04)	0.72 (0.59 to 0.87)	0.75 (0.62 to 0.91)
Other cancer							
Stage I–III	1.0	0.86 (0.84 to 0.88)	0.87 (0.85 to 0.89)	0.92 (0.90 to 0.94)	0.94 (0.92 to 0.96)	0.88 (0.85 to 0.91)	0.91 (0.89 to 0.94)
Stage IV	1.0	0.55 (0.51 to 0.58)	0.55 (0.52 to 0.59)	0.74 (0.69 to 0.80)	0.76 (0.71 to 0.82)	0.63 (0.55 to 0.72)	0.65 (0.56 to 0.74)
Stage unknown	1.0	0.79 (0.77 to 0.81)	0.81 (0.79 to 0.82)	0.84 (0.82 to 0.87)	0.87 (0.85 to 0.90)	0.84 (0.81 to 0.87)	0.88 (0.85 to 0.91)
Eye exams							
Breast cancer							
Stage I–III	1.0	0.88 (0.85 to 0.92)	0.87 (0.83 to 0.91)	1.01 (0.98 to 1.05)	1.00 (0.97 to 1.04)	1.03 (0.97 to 1.08)	1.03 (0.97 to 1.08)
Stage IV	1.0	0.71 (0.57 to 0.88)	0.71 (0.57 to 0.88)	0.96 (0.78 to 1.19)	0.96 (0.77 to 1.19)	0.56 (0.33 to 0.94)	0.55 (0.33 to 0.93)
Stage unknown	1.0	0.92 (0.79 to 1.08)	0.89 (0.76 to 1.04)	0.89 (0.77 to 1.03)	0.87 (0.75 to 1.00)	0.80 (0.65 to 0.98)	0.78 (0.64 to 0.96)
Prostate cancer							
Stage I–III	1.0	1.00 (0.96 to 1.03)	0.97 (0.94 to 1.00)	1.02 (0.99 to 1.05)	0.99 (0.96 to 1.02)	1.09 (1.05 to 1.13)	1.05 (1.01 to 1.09)
Stage IV	1.0	0.93 (0.82 to 1.04)	0.86 (0.77 to 0.97)	1.08 (0.96 to 1.22)	1.02 (0.90 to 1.15)	0.78 (0.61 to 0.99)	0.75 (0.59 to 0.96)
Stage unknown	1.0	1.03 (0.95 to 1.13)	0.97 (0.89 to 1.06)	0.93 (0.86 to 1.01)	0.87 (0.80 to 0.95)	0.98 (0.89 to 1.07)	0.92 (0.83 to 1.01)
Colorectal cancer							
Stage I–III	1.0	0.93 (0.89 to 0.96)	0.91 (0.87 to 0.94)	1.06 (1.02 to 1.09)	1.03 (1.00 to 1.07)	0.99 (0.94 to 1.04)	0.97 (0.92 to 1.02)
Stage IV	1.0	0.58 (0.51 to 0.66)	0.56 (0.49 to 0.64)	0.77 (0.67 to 0.90)	0.77 (0.66 to 0.89)	0.57 (0.40 to 0.80)	0.57 (0.40 to 0.80)
Stage unknown	1.0	0.79 (0.70 to 0.90)	0.76 (0.68 to 0.86)	1.06 (0.95 to 1.18)	1.03 (0.92 to 1.15)	1.02 (0.86 to 1.22)	1.01 (0.85 to 1.20)
Other cancer							
Stage I–III	1.0	0.94 (0.92 to 0.95)	0.92 (0.90 to 0.93)	1.01 (0.99 to 1.03)	1.00 (0.98 to 1.01)	1.03 (1.00 to 1.06)	1.02 (0.99 to 1.04)
Stage IV	1.0	0.72 (0.68 to 0.75)	0.70 (0.67 to 0.73)	0.87 (0.82 to 0.93)	0.86 (0.81 to 0.91)	0.83 (0.74 to 0.93)	0.83 (0.74 to 0.92)
Stage unknown	1.0	0.95 (0.93 to 0.97)	0.92 (0.90 to 0.94)	0.98 (0.95 to 1.00)	0.95 (0.92 to 0.97)	0.92 (0.89 to 0.96)	0.90 (0.87 to 0.93)

* ARR = adjusted relative rate (adjusted for age, sex, diabetes duration, weighted aggregated diagnosis group score, neighborhood income quintile, and rurality);

CI = confidence interval; URR = unadjusted relative rate.

## Discussion

This population-based study documented a worrisome rise in preventable diabetic complications among diabetes patients following a diagnosis of cancer. Compared with diabetes patients without cancer, we found a statistically significantly higher rate of hospital visits for diabetic emergencies, skin and soft tissue infections, and cardiovascular events in the first year after a cancer diagnosis for all major cancer types except stage I–III prostate cancer. Given that these complications are potentially preventable with appropriate outpatient diabetes management (20), our findings highlight a vulnerable health care period in which diabetes care needs are not being adequately met.

The strengths of our study include its large population-based nature in a universal health care setting, which increases generalizability and eliminates access barriers as potential mediators of the findings. We had complete data on hospitalizations for clinical outcomes and validated registries to identify cases of diabetes and cancer, and we were able to isolate cancer subtypes and stage to address differences in cancer treatment and prognosis. There were, however, limitations. First, though the databases we used were robust, some of the cancer types and stages within the registries may have been misclassified. Second, while we adjusted for several confounders, we did not include potential mediators such as glycemic control, medication adherence, and cancer treatments. Third, our ability to evaluate diabetes-related management was limited to primary care visits for diabetes and eye examinations, which under-represents the full spectrum of diabetes care. Fourth, our study population was limited to patients with diabetes; it is uncertain how these risks compare with cancer patients without diabetes. Finally, as in all observational studies, we cannot exclude a selection bias due to unmeasured confounders. We minimized this risk by defining cancer exposure as a time-varying covariate, which allowed patients to contribute time to both cancer and noncancer exposure.

One other study has evaluated diabetic complications after a cancer diagnosis. Using the UK Clinical Practice Research Datalink, Griffiths et al. found no difference in long-term microvascular and macrovascular outcomes between diabetes patients with and without cancer (19). However, they did not consider short-term complications as the first year after cancer diagnosis was excluded in that study. In contrast, our study focused specifically on the period after a cancer diagnosis. That period correlates with a stressful time when patients may have demanding cancer care regimens (25), and attention is often diverted away from diabetes management to focus on cancer treatment. Some studies have examined the processes of diabetes care in the postcancer period. In one study, colorectal cancer patients with diabetes were significantly less likely than noncancer controls to attend regular diabetes visits and annual eye exams after cancer diagnosis (12). Another study found that the frequency of HbA1c measurements decreased in patients after diagnosis with colorectal and breast cancer (13). Cancer patients in general are less likely to see their primary care physician during the adjuvant treatment phase (26), a finding that was confirmed in our study. Interestingly, two studies conducted within regional integrated health care systems contrast these findings, showing higher frequency of diabetes visits and HbA1c measurements and better glycemic control in patients with cancer when compared with controls (14,15). Diabetes care may have been more compromised in our more generalized study setting, whereby specialized cancer care is most often provided separate from patients’ usual care team. Even if diabetes monitoring and visits are deferred during the active cancer treatment period, ongoing support for diabetes control should nonetheless be provided so that more serious complications can be averted in this already vulnerable population.

Not surprisingly, the impact of a cancer diagnosis on diabetic complications differed depending on the type and stage of cancer. For instance, the risk of complications was substantially higher for all stage IV cancers, likely reflecting their higher comorbidity and decreased intensity of medical care. This is corroborated by the decrease in diabetes-related process indicators after a stage IV cancer diagnosis, which may be appropriate in this setting. We also found that diabetic emergencies and cardiovascular events were highest after a colorectal cancer diagnosis, which may be due to greater risks associated with the more intensive surgical and chemotherapeutic regimens. Interestingly, complication rates were not increased in patients with diabetes who developed stage I–III prostate cancer. These findings are consistent with those of another study, which found decreased adherence to diabetes medications in all patients after a cancer diagnosis except for those with prostate cancer (18). These findings may reflect differences in the natural history and management of the disease. Prostate cancer is generally associated with a relatively good prognosis, where up to 45% of patients are followed with an active surveillance strategy rather than receiving cancer treatment (27,28). A prostate cancer diagnosis may therefore have less of an impact on diabetes management, and patients are less likely to be exposed to cancer treatments that could disrupt glycemic control. Indeed, the decrease in visits for diabetes management after cancer was negligible in patients with stage I–III prostate cancer.

Our study highlights a period in which diabetes patients may be particularly vulnerable to disruption of diabetes control and acute complications. First, cancer surgery and adjuvant treatment increase the risk of metabolic stress and glycemic decompensation (29). Second, intensive chemotherapy regimens may distract from adequate diabetes management (16–18). Third, many anticancer drugs and glucocorticoids, which are a common adjunct to many chemotherapeutic regimens, are known to induce hyperglycemia (30,31). Hyperglycemia places patients at higher risk for peri-operative wound infections (32,33), cellulitis after breast-conserving therapy (34), and cardiovascular events (35,36). Lower adherence to cardio-protective medications (16,17), postoperative cardiac complications (37), and radiation-induced cardiotoxicity (34, 35) may further contribute to cardiovascular events during this period. A rise in cardiovascular deaths in the early period after cancer has also been attributed to the stress associated with a cancer diagnosis (38). As diabetes is an ambulatory-sensitive condition, hospitalizations for diabetic complications are potentially preventable with effective diabetes monitoring and treatment (20). These findings thus demonstrate a need for more enhanced outpatient diabetes care in the postcancer period. Patients may not always have timely access to a diabetes care team, and resources and expertise to comanage diabetes may be limited within cancer centers. There may also be a lack of care coordination and poor communication during transitions between their oncology, diabetes, and primary care teams. Health care delivery models that better integrate diabetes and cancer treatment are necessary to optimize care and prevent hospitalizations for complications.

In conclusion, we showed a significant rise in acute diabetic complications after a diagnosis of cancer in patients with diabetes. These findings have important implications for the burden of diabetes on health care systems and patients, especially as the population of patients with cancer and comorbid diabetes continues to increase (1). Patients with diabetes already have a higher risk of cancer and poorer survival (2,3); a rise in diabetic complications not only adds to their overall morbidity but can further worsen their cancer prognosis (5,6). As these outcomes are potentially preventable with appropriate outpatient services, there is a clear need to improve the integration of cancer treatment with ongoing delivery of diabetes care. Further research to test appropriate health care interventions is needed to improve morbidity and mortality from both diabetes and cancer.

## Funding

This work was supported by a Canadian Institutes of Health Research (CIHR) operating grant (MOP#123263) and by the Ontario Institute for Cancer Research and Cancer Care Ontario.

Dr. Lipscombe was supported by a CIHR New Investigator Award. Dr. Peter Austin is supported in part by a Career Investigator award from the Heart and Stroke Foundation. This study was conducted with support from a CIHR operating grant (MOP#123263) and by the Ontario Institute for Cancer Research and Cancer Care Ontario. This study was supported by the Institute for Clinical Evaluative Sciences (ICES), which is funded by an annual grant from the Ontario Ministry of Health and Long-Term Care (MOHLTC). The opinions, results, and conclusions reported in this paper are those of the authors and are independent of the funding sources. No endorsement by ICES or the Ontario MOHLTC is intended or should be inferred. 

## Notes

Affiliations of authors: Department of Medicine (EW, MKK, LLL), Institute for Clinical Evaluative Sciences (KF, HDF, PCA, MKK, LLL), and Institute of Health Policy, Management and Evaluation (PCA, MKK, LLL), University of Toronto, Toronto, ON, Canada; Department of Medical Oncology, Princess Margaret Hospital, Toronto, ON, Canada (MKK); Department of Medicine, Women’s College Research Institute, Women’s College Hospital, Toronto, ON, Canada (LLL).

Parts of this material are based on data and/or information compiled and provided by the CIHI. However, the analyses, conclusions, opinions, and statements expressed in the material are those of the authors, and not necessarily those of CIHI. Parts of this material are based on data and information provided by Cancer Care Ontario (CCO). The opinions, results, view, and conclusions reported in this paper are those of the authors and do not necessarily reflect those of CCO. No endorsement by CCO is intended or should be inferred. We thank ServiceOntario for use of Office of the Registrar General (ORG) information on deaths. The views expressed herein do not necessarily reflect those of ORG or the Ministry of Government Services. The authors have no conflicts of interest to declare. The authors would like to thank Christina Yu for assistance with manuscript preparation and submission.

Erin Worndl was the primary author of the paper, contributing to the conception and design of this project. She was responsible for drafting the article and its revisions. She approved the final version to be published. Hadas Fischer assisted with the conception of this project and ensuring that it was in line with the guidelines for research mandated by the Institute for Clinical Evaluative Sciences. She also assisted with revision of this article, especially with respect to methodology. She approved the final version to be published. Kinwah Fung assisted with the design of this project and conducted all data analyses. She assisted with revising the article, especially with respect to the Methods and Results sections and ensuring accuracy of all tables and figures. She approved the final version to be published. Monika Krzyzanowska contributed to the concept and design of this project and to the article revisions. She approved the final version to be published. Peter Austin assisted with the conception and design of this project and with the revision of the manuscript. He approved the final version to be published. Lorraine Lipscombe supervised this project and was heavily involved in its conception and design. She assisted with editing all versions of the manuscript and has approved the final version to be published.
